# 

**Published:** 1874-09

**Authors:** 


### ACKNOWLEDGMENTS.

Atlanta Daily Herald; Rome Daily Commercial; The Commonwealth; American Journal of Pharmacy; American Practitioner; Archives of American Medicine and Surgery; Boston Medical and Surgical Journal; Baltimore Physician and Surgeon; Buffalo Medical and Surgical Journal; The Clinic; The Druggist; The Galaxy; Herald of Health; Journal of Materia Medica; London Lancet; Louisville Medical Reporter; Medical Record; Medical News and Library; Medical and Surgical Reporter; Medical Times; Nashville Journal of Medicine and Surgery; Pacific Medical and Surgical Journal; Popular Science Monthly; Physician and Pharmacist; Supplement to Medical News and Library; Texas Medical Journal; Virginia Medical Monthly.

